# Impact of Health All-in-One Machines on access to healthcare of rural areas in China: an interrupted time series analysis

**DOI:** 10.1186/s12913-025-12710-z

**Published:** 2025-04-12

**Authors:** Yan Xie, Hanning Zhang, Wenqi Li, Hui Yan, Huilong Duan

**Affiliations:** 1 State Key Laboratory of Digital Medical Engineering, Key Laboratory of Biomedical Engineering of Hainan Province, School of Biomedical Engineering, Hainan University, Sanya, Hainan 572024 China; 2 School of Information and Communication Engineering, Hainan University, Haikou, Hainan 570228 China; 3 China Unicom (Hainan) Innovation Research Institute, Haikou, 570100 China; 4 China Unicom (Hainan) Industrial Internet Co., Ltd, Haikou, 570100 China; 5 College of Biomedical Engineering and Instrumental Science, Zhejiang University, Hangzhou, Zhejiang 310027 China

**Keywords:** Smart healthcare systems, Access to healthcare, Health all-in-one machines, HAMs intervention, ITS study, ARIMA models

## Abstract

**Background:**

Smart healthcare systems are expected to have a positive impact on addressing challenges in healthcare. However, the real-world adoption and widespread integration of Smart healthcare systems still face many barriers, and their clinical utility lacks empirical research with large sample sizes, particularly in rural areas. The aim of this study is to evaluate the impact of a new smart healthcare system, the Health All-in-One Machines (HAMs), on improving the health services in rural areas of China.

**Methods:**

The data included health services information from 1,866 village clinics in Hainan, China, covering the period November 30, 2020, to April 30, 2023. The impact of Health All-in-One Machines on access to healthcare was measured using four outcome indicators: the number of patient visits, medical revenue, pharmaceutical revenue, and medical expense per patient. We conducted a three-phase interrupted time series study to explore the effects of the Health All-in-One Machines intervention on these indicators across two distinct periods: the second phase (26 weeks, adaptation period) and the third phase (74 weeks, full-scale implementation period).

**Results:**

The interrupted time-series analysis revealed that the Health All-in-One Machines intervention had no significant impact on outcome indicators comparing the pre-intervention period to the adaptation period. However, from the adaptation period to full implementation, significant impacts were observed. Specifically, notable level changes were observed: the number of patient visits increased by 37.85% (*p* < 0.01), medical revenue increased by 54.03% (*p* < 0.001), pharmaceutical revenue increased by 32.84% (*p* < 0.05), and medical expense per patient increased by 2.368 CNY (*p* < 0.001). Additionally, a significant trend change was observed in medical expense per patient, with a decrease of 0.15 CNY per week (*p* < 0.05).

**Conclusions:**

This study provides empirical evidence of some positive changes in the Health All-in-One Machines intervention on the outcome indicators regarding the access to healthcare. Moreover, our analysis indicates that the Health All-in-One Machines intervention would at least take longer to take effect when implemented in large-scale rural healthcare institutions. The findings from this study provide insights for future delivery and policy making of Smart healthcare systems in rural areas.

**Supplementary Information:**

The online version contains supplementary material available at 10.1186/s12913-025-12710-z.

## Background

Smart healthcare systems (SHSs) are technology-driven systems that utilize advanced technologies like Artificial Intelligence (AI), Internet of Things (IoT), big data analytics, and other digital tools to improve healthcare services [1–3]. Health All-in-One machines (HAMs) are a type of SHSsthat integrate multiple health monitoring and diagnostic functions into a single unit [4, 5]. On the one hand, HAMs could replace basic medical equipments by integrating multiple vital signs tests, and adding enhanced features like electrocardiograms, urine analysis, and fetal heart rate monitoring; on the other hand, advanced functionalities such as cloud storage, telemedicine, and AI-assisted diagnoisis could also be incorporated to support primary doctors in improving diagnosis accuracy and treatment effectiveness [6, 7]. By combining these systems into a single platform, the HAMs offer a comprehensive solution tailored to meet the health service needs of communities and rural areas.

Access to healthcare, one of the central for the performance of health care systems [8], is defined as the timely use of personal health services to achieve the best health outcomes [9]. Rural residents continue to face significant obstacles in accessing adequate medical services. These obstacles include inadequate medical resources, outdated equipments, physician shortages, weak health awareness and other disparities [10, 11]. Improving access to healthcare is a crucial step in enhancing the capability of health services in rural areas. This capability improvement has the potential to reduce mortality and morbidity rates related to chronic diseases and common diseases in rural regions. The number of patient visits and medical revenue are two measures that reflect the capability of health services [12, 13].

Globally, HAMs market has witnessed significant growth [4, 5]. Given the increasing demand for HAMs in rural healthcare settings, more evidence is required to assess their impact. As an extended function within HAMs, telemedicine capabilities have demonstrated significant improvement in access to healthcare. In 2018, Natafgi et al. confirmed that telemedicine has the potential to reduce the number of patient transfers and generate more revenue for rural hospitals [14]. In 2020, Xu et al. conducted a study that confirmed telemedicine would increase the utilization of outpatient and inpatient services [15]. However, studies on the practical impacts of HAMs on access to healthcare in rural areas are sparse, especially quantitative evidence for large sample sizes [16, 17]. Thus, this study aims to find the evidence of the HAMs intervention on access to healthcare using real-world data from a large amount of village clinics.

The Primary Healthcare Capability Promotion Project was initiated in December 2020 by the Hainan government. The overall goal of this project is to improve the quality of primary health services through the combination of AI units and 5G IoT devices. This project has covered 2,700 village clinics and 340 township health centers across the province, with a budget exceeding 200 million CNY. As a key component of this project, the HAMs have been successfully installed in these village clinics, with their cumulative usage exceeding 882,000 times as of April 2023. To explore whether the HAMs can improve access to healthcare, an interrupted time series (ITS) study was used to evaluate the changes in the number of patient visits, medical revenue, and medical expense per patient. To address the common issues of seasonality, autocorrelation, and nonstationarity in time series, we adopted the autoregressive integrated moving average (ARIMA) models. Considering the varying response levels among village clinics and the inconsistencies introduced by the large-scale intervention, we implemented a three-phase ITS design to better identify the impact of HAMs intervention over different time periods. This study provides valuable insights for enhancing the implementation of HAMs in rural areas.

## Methods

### Study setting

This study was conducted in Hainan, China, a tropical province located in the southernmost part of the country. According to the 2020 census, Hainan has a rural population of 4 million [18]. There are 2764 village clinics and 8344 staff members according to the 2021 Hainan Provincial Health Statistics Yearbook [19]. These clinics serve over 5.73 million outpatient visits annually. They face common challenges seen in rural areas across China, such as limited medical resources, uneven distribution of healthcare personnel, and low operational efficiency.

### Study design

This study employed a retrospective, pre-post uncontrolled quasi-experimental design to assess the impact of HAM implementation on healthcare access in village clinics across Hainan Province. All data for this study were sourced from the primary health information system (PHIS) in Hainan, which consolidates and stores the electronic health records of patients in provincial village clinics. The PHIS system was introduced in the second half of 2020; however, an analysis of its database indicated that data on the number of patient visits were only available until November 2020. Therefore, the starting date of this study period was defined as November 30, 2020. The end date was set as the time we collected the data, which was April 30, 2023.

Given that users require sufficient time to become familiar with the HAMs, their effects may take time to fully emerge. The implementation of large-scale HAMs intervention may introduce further complexity, making it unrealistic to assume that the intervention was fully implemented at a single point in time [20]. Therefore, a three-phase ITS design was used to evaluate the longitudinal impacts of HAMs on access to health services. The first phase was designated as the pre-intervention phase, spanning 26 weeks from November 30, 2020, to May 30, 2021. During this period, HAMs were newly deployed to village clinics, and testing and training sessions were mainly carried out. At that time, the average usage of HAMs was approximately one per week according to the data we collected. The second phase marked the adaptation period, spanning 26 weeks from May 31, 2021, to November 28, 2021. During this period, village clinics began adopting HAMs in their daily use. The third phase, spanning 74 weeks from November 29, 2021, to April 30, 2023, marked the full-scale implementation period, during which HAMs became fully operational. Village clinics that both implemented the HAMs intervention and had records of patients’ visits using HAMs were selected as the study subjects.

### Outcome measures

Access to healthcare plays a pivotal role in promoting and maintaining health, as well as preventing and managing diseases [21]. As the ultimate objective of numerous health reforms, access to healthcare can be improved by optimizing health services, focusing on convenience, timeliness, and efficiency. Considering the functional role of village clinics, this study examined the following outcome indicators: the number of patient visits, medical revenue, pharmaceutical revenue, and medical expense per patient.

The number of patient visits serves as a direct indicator of healthcare service utilization, reflecting how HAMs improve access to healthcare for rural residents. Medical revenue captures both the financial performance and service capability of village doctors, while pharmaceutical revenue highlights the availability and use of medications, reflecting their role in supporting comprehensive and timely care. Finally, medical expense per patient provides valuable insights into the affordability and cost-efficiency of healthcare.

These indicators offer a comprehensive evaluation of HAMs’ role in addressing access challenges and enhancing the capability of health services in rural areas.

### Three-phase ITS analysis

Data on outcome indicators were collected from electronic health records spanning November 30, 2020, to April 30, 2023. After removing the village clinics with no records of patient visits, 1,866 village clinics were included in the study. During the data preprocessing phase, patient visit records with unusual medical expense (pharmaceutical revenue exceeding 400, which is above the 99.9th percentile) were excluded, resulting in a final dataset of 2,957,936 records. Subsequently, we analyzed the daily health service data and aggregated it on a weekly basis, resulting in a time series for each outcome indicator with 126 observation points.

To analyze the correlation between intervention and outcome, we adopted a three-phase ITS design using ARIMA models, a widely recognized approach for evaluating effects. The ITS analysis is based on the hypothesis that, in the absence of an intervention, outcomes would continue to follow the same level and trend as observed during the pre-intervention period—referred to as the “counterfactual” [22]. By comparing the actual post-intervention data with this counterfactual trend, the effect of the intervention can be estimated. ITS design is susceptible to the influence of time-varying confounders that change very rapidly, such as a specific infectious disease prone to outbreaks [22]. Therefore, we introduced a time-varying control variable that accounted for the impact of the Spring Festival holiday and the COVID-19 outbreak period in Hainan. During this period, many village clinics were temporarily closed, which had a negative impact on access to healthcare.

The following three-phase regression model was applied to assess the impact of HAMs intervention:

1 $$\begin{aligned}\:{Y}_{t}=&{\beta}_{0}+{\beta}_{1}\:Tim{e}_{t}+{\beta}_{2}{x}_{t}^{6 m}+{\beta}_{3}{x}_{t}^{12 m}\\&+{{\beta}_{4}\:Time}_{t}^{6 m}+{{\beta}_{5}\:Time}_{t}^{12 m}\\&+{{\beta}_{6}x}_{t}^{con}+{n}_{t}\end{aligned}$$

where the dependent variable *Y*_*t*_ is an aggregated outcome in week *t*. Independent variables consist of six time-related variables as follows: $\:Tim{e}_{t}$ is a time variable from the start to the end of the study ($Tim{e}_{t}$ = 1,2,…,126). Two binary variables related to the period, $\:{x}_{t}^{6 m}$ was coded as 0 during the first phase and as 1 in the remaining two phases; $\:{x}_{t}^{12 m}$ was coded as 0 during two early phases and as 1 in the third phase. $\:{Time}_{t}^{6 m}$ and $\:{Time}_{t}^{12 m}$ are variables counting the number of weeks after the first phase and second phase respectively. They were used to estimate the changes in trend or level along with time. $\:{x}_{t}^{con}$ represents an observable time-varying control variable.

In Eq. (1), the coefficient $\:{\beta}_{0}\:$is the regression intercept; the coefficient $\:{\beta}_{1}$ estimates the trend of outcome indicators within the first phase; $\:{\beta}_{2}$ and $\:{\beta}_{3}$ estimate the immediate level changes of outcome indicators that occur in the second and third phases, respectively; $\:{\beta}_{4}$ and $\:{\beta}_{5}$ estimate the difference in trends between the first phase and second phases of intervention, and the difference in trends between the second and third phases of intervention, respectively; $\:{\beta}_{6}$ estimates a regression coefficient of the control variable.

The error $\:{n}_{t}$ is an ARIMA model [23], that is:

2 $$\begin{aligned}\:{\varDelta}^{d}{n}_{t}=&{\phi}_{1}{\varDelta}^{d}{n}_{t-1}+\dots\:+{\phi}_{p}{\varDelta}^{d}{n}_{t-p}\\&+{\theta}_{1}{z}_{t-1}+\dots\:+{\theta}_{q}{z}_{t-q}+{z}_{t}\end{aligned}$$

where the parameters *p*, *d* and *q*, represent the autoregressive order, the differencing order, and the moving average order, respectively [24]. The regression model with ARIMA errors is used to account for autocorrelation and nonstationarity of the residuals in ITS analysis.

Outliers in the outcome indicators were identified using the BoxPlot-based method, and these outliers were replaced by employing staged averages. To stabilize variance, log transformation was applied to certain outcome indicators with large values, including the number of patient visits, medical revenue, and pharmaceutical revenue. The optimal parameters for the ARIMA errors model were determined using the *auto.arima* function in R, which combines unit root testing, minimization of the Akaike Information Criterion (AIC), and Maximum Likelihood Estimation (MLE). For each regression model, autocorrelation of the residuals was detected using the autocorrelation function (ACF) and partial autocorrelation function (PACF). The Ljung–Box test was applied to assess the randomness of residuals. To assess the accuracy of the model’s predictions (counterfactuals), we examined how well the pre-intervention data fit the selected model. Specifically, we employed measures such as Residual Variance (RV), Root Mean Squared Error (RMSE), Mean Absolute Percentage Error (MAPE) to quantify the model’s goodness-of-fit. A *p* value < 0.05 was considered to indicate statistical significance. Statistical analysis was performed using R version 4.2.1.

## Results

### Data descriptive analysis

The study period was divided into three phases: the first phase for the intervention (26 weeks, pre-intervention period), the second phase for the intervention (26 weeks, adaptation period) and the third phase for the intervention (74 weeks, full-scale implementation period). The patient data from each village clinic were reviewed throughout the study period. The data were organized according to the four outcome indicators. Time series for each indicator were generated from a cohort of 1,866 village clinics and categorized into 126 weeks.

We adopted the BoxPlot method to identify outliers in each phase of the data. The analysis revealed a total of 7, 6, 8 and 1 outliers for the number of patient visits, medical revenue, pharmaceutical revenue, and medical expense per patient respectively. These outliers were associated with the Spring Festival and the COVID-19 outbreak. After processing the anomalous observations, Table 1 presents the weekly averages of the outcome indicators across three phases.

**Table 1:** The statistical analysis of outcome indicators in three phases

Outcome indicators	The first phase	The second phase	The third phase
The number of patient visits	19594.80	19022.76	25843.29
Medical revenue (CNY)	556703.56	551032.18	838246.87
Pharmaceutical revenue (CNY)	362472.08	366603.99	481175.49
Medical expense per patient (CNY)	28.38	29.02	32.81

As shown in Table 1, during the second phase of the HAMs intervention, there were no significant changes in outcome indicators related to access to healthcare compared to the first phase. However, there were significant differences between the first phase and third phase of the HAMs intervention for all outcome indicators. That is, the averages of the outcome indicators increased.

**Fig. 1:**
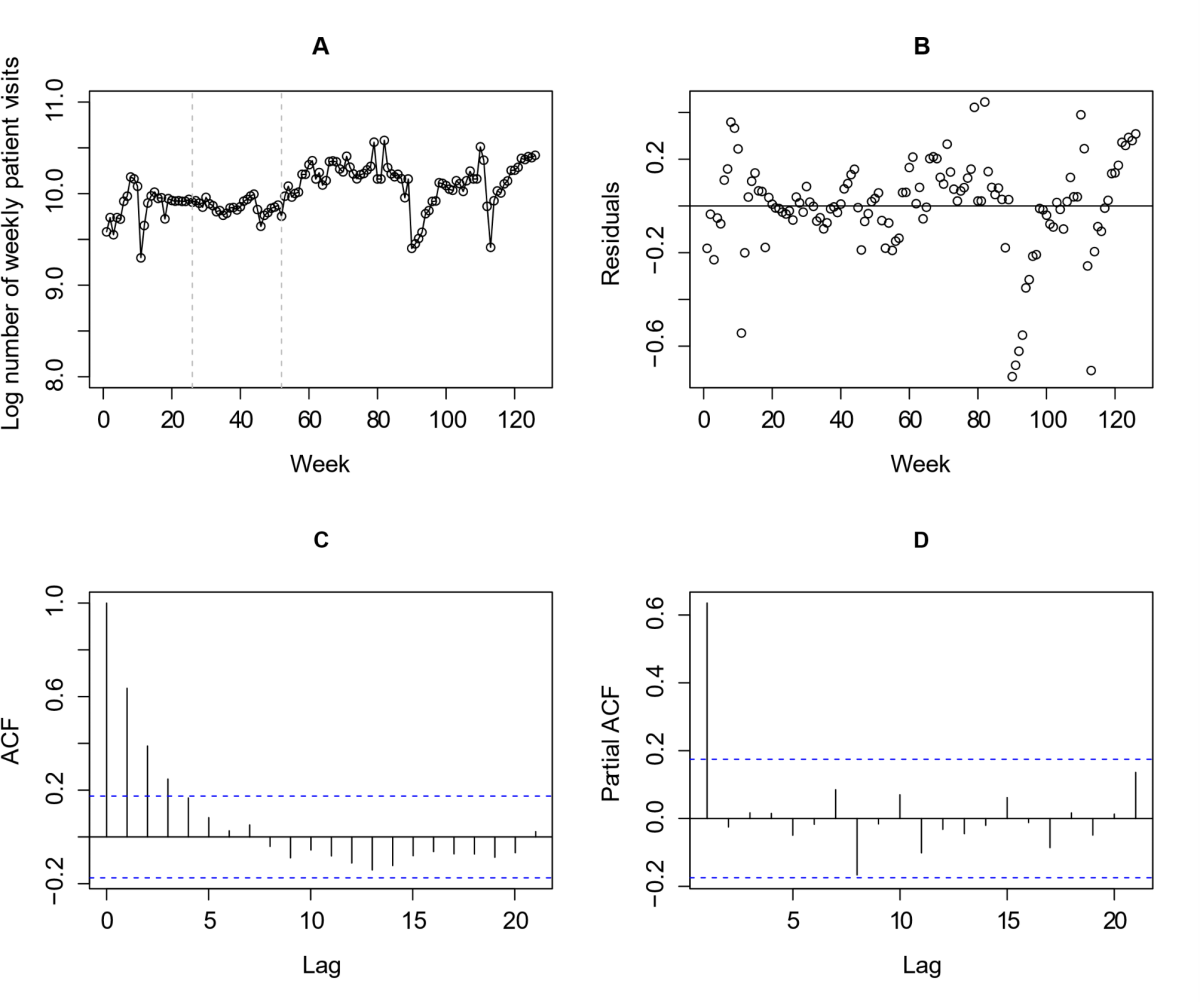
Time series analysis of the number of patient visits: the time series of the number of patient visits (**A**), the residuals of segmented regression with no ARIMA errors modeling (**B**), autocorrelation function for the residuals (**C**), and partial autocorrelation function for the residuals (**D**)

The time series data, consisting of weekly patient visits, are presented in Fig. 1A. When we fitted a segmented linear regression model with no ARIMA errors, the residual plot, along with ACF and PACF plots of the residuals, are shown in Fig. 1B, C and D, respectively. These plots indicate that the independent residuals assumption is unreasonable for these data. In the ACF plot (Fig. 1C), we observed a significant autocorrelation that gradually dies off at lag 5. However, the PACF plot (Fig. 1D) shows that the autocorrelation is primarily explained by lag 1. The analyses for time series remaining three outcome indicators are shown in the Supplementary Materials Fig. S1-S3. The observed residual autocorrelation, following the segmented linear regression model, supports our decision to use ARIMA modeling.

### Effects of the HAMs intervention on access to healthcare

Based on Eqs. 1 and 2, we have adopted a three-phase ITS design to examine the changes in outcome indicators associated with village clinics as the intervention period of HAMs progressed.

#### Indicator 1: the number of patient visits

**Fig. 2:**
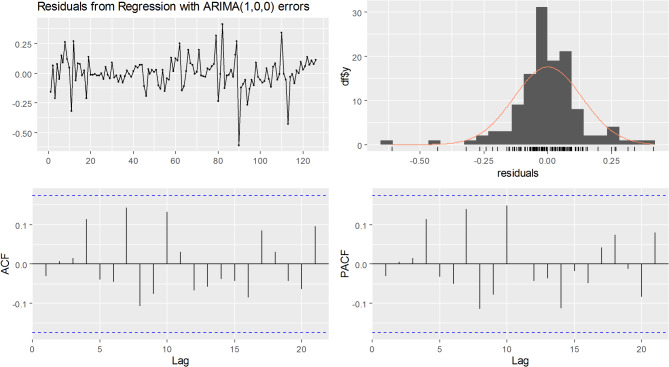
Residual check of the number of patient visits for final ARIMA (1,0,0)

The *auto.arima* function from the *forecast* package in R was employed to automatically determine the optimal ARIMA model with the lowest information criterion. Time-related variables were used as external covariates to estimate the intervention effects. After an iterative search, the best model was ARIMA(1,0,0), indicating that this model is an autoregressive (AR) model with a lag of 1. The residual plots are shown in Fig. 2, where no discernible patterns or significant autocorrelation can be observed in the residuals. The *P*-value for the Ljung-Box test was 0.726, providing support for the hypothesis that the residuals follow a white noise distribution. Consequently, our selected model exhibited a good fit.

**Fig. 3:**
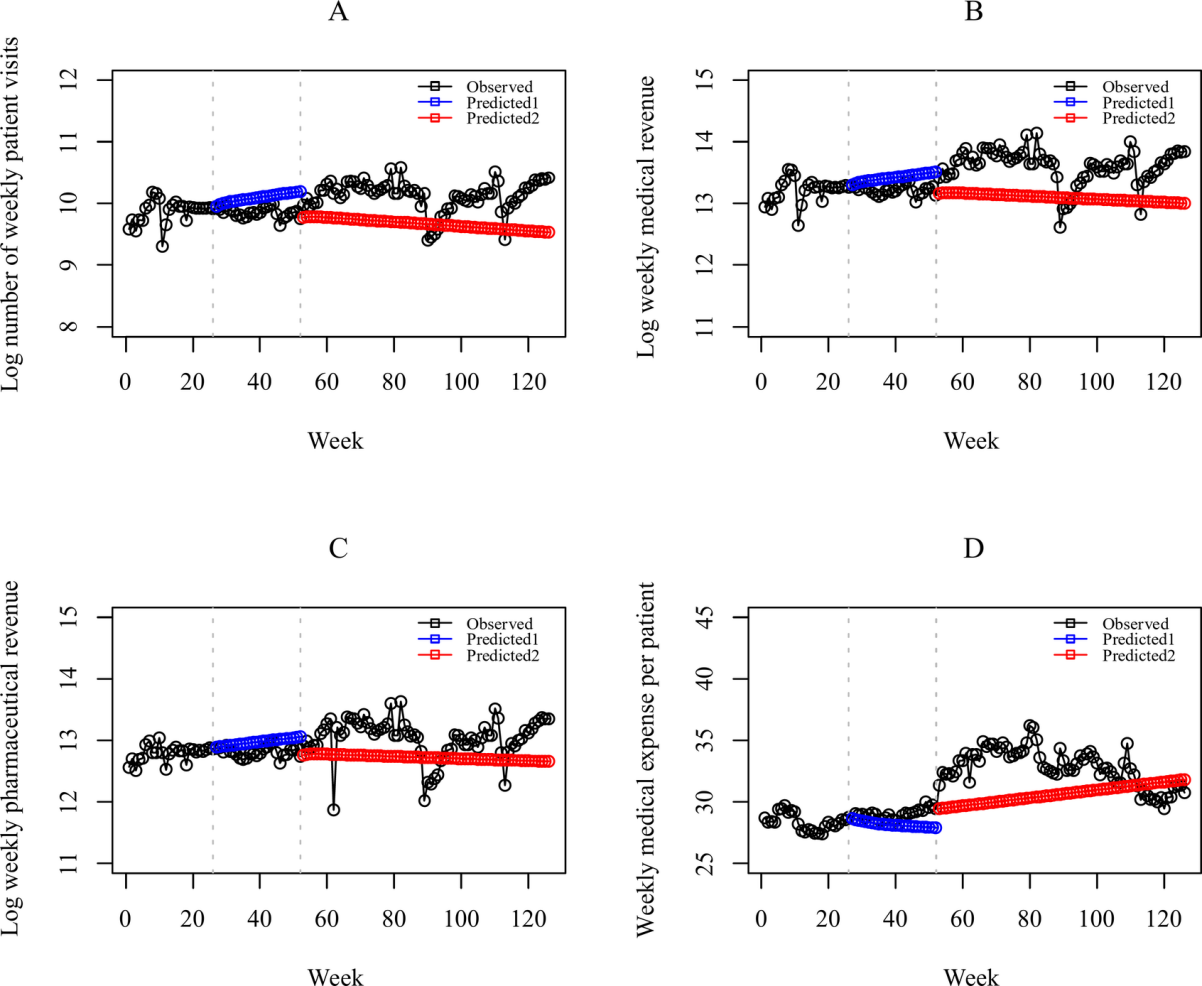
Observed values and predicted values in absence of intervention based on ARIMA model

Figure 3A illustrates the ITS analysis for the number of patient visits in all village clinics. The X-axis depicts time (in weeks), with the timeline was divided into three HAMs intervention phases by vertical lines at weeks 26 and 52. The comparison between the values predicted by our model in the absence of the intervention (counterfactual) and the observed values is plotted in Fig. 3A. We observed obvious difference between the two in the third intervention phase. The corresponding coefficients of the model are presented in Table 2 (columns 2–3). In this table, we can see that the first phase of the HAMs intervention showed a very small increasing trend (0.007% point increase per week, 95% CI − 0.006 to 0.021), but this result was not statistically significant (*p* = 0.285). Compared to the first phase, no statistically significant changes were observed in either the level or trend of patient visits during the second intervention phase. The observed phenomenon can be attributed to the implementation of HAMs in 2,700 village clinics, coupled with a slower rollout in remote areas. Consequently, the effects of the HAMs intervention may require more time to manifest than intervention implemented in single medical institutions or in well-developed regions. The regression results support this hypothesis, indicating that the level change in the number of patient visits significantly increased by 37.85% ($\:{=exp}^{0.321}-1$, *p* = 0.003) during the third phase of the intervention, while the trend for the number of patient visits remained fairly stable in this phase.

#### Indicator 2: the medical revenue

The impact of the HAMs introduction on total weekly medical revenue is displayed in Fig. 3B, which also shows the difference between the observed values and the counterfactual predictions. The model selected by the iterative search algorithm was ARIMA(1,0,0), and the Ljung-Box test (*p* = 0.419) supported that the autocorrelation of residuals was appropriately addressed by the ARIMA errors model. The residual check for this model is presented in the Supplementary Materials Fig. S4. The corresponding regression results are presented in Table 2 (columns 4–5). For total weekly medical revenue, we observed a pattern of effects similar to that observed for the number of patient visits. While no changes were detected during the second phase of the intervention, significant changes were observed during the third phase. The weekly medical revenue of village clinics also experienced an immediate and significant increase by 54.03% ($\:{=exp}^{0.432}-1$, *p* < 0.05), and the trend change continued unbroken.

**Table 2:** Model parameter estimates from the three-phase ITS

Parameter	The number of patient visits		Medical revenue		Pharmaceutical revenue		Medical expense per patient	
	Coefficients	*P*-value	Coefficients	*P*-value	Coefficients	*P*-value	Coefficients	*P*-value
Initial level	9.774 (0.113)	< 0.001	13.137 (0.121)	< 0.001	12.760 (0.111)	< 0.001	28.744 (1.042)	< 0.001
Initial trend	0.007 (0.007)	0.285	0.006 (0.007)	0.392	0.005 (0.006)	0.450	− 0.021 (0.059)	0.727
Level chang after HAM- 2^a^	− 0.059 (0.124)	0.636	− 0.060 (0.132)	0.653	− 0.072 (0.137)	0.601	− 0.193 (0.616)	0.753
Level chang after HAM- 3^b^	**0.321** (0.110)	< 0.01	**0.432** (0.120)	< 0.001	**0.284** (0.116)	< 0.05	**2.368** (0.638)	< 0.001
Trend chang after HAM- 2	− 0.010 (0.010)	0.344	− 0.006 (0.011)	0.580	− 0.0057 (0.010)	0.576	0.144 (0.096)	0.131
Trend chang after HAM- 3	0.003 (0.007)	0.725	− 0.0008 (0.007)	0.912	0.0009 (0.007)	0.897	-**0.150** (0.060)	< 0.05
Control variable	− 0.370 (0.054)	< 0.001	− 0.416 (0.060)	< 0.001	− 0.627 (0.065)	< 0.001	− 0.951 (0.222)	< 0.001
AR(1)	0.581 (0.077)	< 0.001	0.580 (0.080)	< 0.001	0.426 (0.082)	< 0.001	0.850 (0.050)	< 0.001
Box-Ljung test	--	0.726	--	0.419	--	0.600	--	0.550

HAM-2^a^ : the second phase of the HAMs intervention

HAM-3^b^ : the third phase of the HAMs intervention

#### Indicator 3: the pharmaceutical revenue

The analysis results for pharmaceutical revenue are consistent with the previous two evaluation conclusions. No significant changes in the level or trend were observed during the second intervention phase compared to the first phase. However, a significant change in the level was identified during the third intervention phase. Figure 3C illustrated the difference between actual pharmaceutical revenues and predicted values. The results of the regression analysis based on the ARIMA errors model are presented in Table 2 (columns 6–7), with the residual check provided in the Supplementary Materials Fig. S5. Following the full-scale implementation of the HAMs intervention, which spanned a period of more than one year, we observed a statistically significant increase by 32.84% ($\:{=exp}^{0.284}-1$, *p* < 0.05) in the weekly pharmaceutical revenue generated by village clinics.

#### Indicator 4: the medical expense per patient

When analyzing the impact of the HAMs intervention on medical expense per patient, we decided not to apply a logarithmic transformation to this outcome variable due to its relatively small value, which remained at approximately 30. The scatter plot of medical expense per patient over time and the counterfactual predicted values for the second and third intervention phases are shown in Fig. 3D. The regression results are presented in Table 2 (columns 8–9), indicating a significant increase in the level change by 2.368 CNY (*p* < 0.001) and a decrease in the trend change by 0.150 CNY (*p* < 0.05) during the third phase for the intervention. The residual check in Supplementary Materials Fig. S6 showed independent residuals and confirmed the model’s adequacy. These findings suggest that there was a deceleration in the upward trend of medical expense per patient as HAMs became operational and optimized.

 Supplementary Materials Table S1 presented the measures of ARIMA(1,0,0) error models’ fit for the first-phase data, while Table S2 showed the results for the second-phase data. The evaluation results demonstrated the models’ ability to fit the pre-intervention data, as indicated by lower VR, RMSE, and MAPE values. These findings confirmed the reliability of our selected ARIMA(1,0,0) error models and supported the credibility of our effect analysis.

## Discussion

To the best of our knowledge, this study represents the first empirical evaluation of the HAMs intervention. This study investigated the impact of the HAMs intervention (comprising the integration of multimodal health monitoring, AI-aided diagnosis, and telemedicine) on access to healthcare in large-scale village clinics located in Hainan Province, China. Improvements in access to healthcare can be observed through indicators such as the number of patient visits, medical revenue, pharmaceutical revenue, and per patient medical expense, etc. Considering the implementation background of HAMs intervention, a quasi-experimental study based on three-phase ITS models was designed to explore the real-world effects of the HAMs intervention. This study provides evidence of whether HAMs intervention in rural areas improves the quality of care.

Our findings demonstrated that the impact of HAMs intervention on the number of patient visits. Specifically, we observed a significant increase of 37.85% in the number of patient visits following the full-scale implementation of HAMs. This finding aligns with existing literature, which showed that digital health products sharing similar features with HAMs wereeffective in primary healthcare settings. A systematic review showed that digital health strategies increased frequency of people seeking care in primary healthcare facilities during the COVID- 19 pandemic, especially in remote areas with difficult access and little face-to-face demand [25]. Similarly, a quasi-experimental study found that integrated health information systems for primary healthcare significantly improved patients’ healthcare-seeking behavior and enhanced treatment adherence [26]. The HAMs can provide multimodal health detection capabilities, and the integrated design facilitates comprehensive assessments of patients’ health status. Moreover, the HAMs facilitate convenient management of patient health information and enable remote interaction with higher-level medical institutions. These functions empower health workers to enhance their treatment capabilities, thereby reducing the need for patient transfers and improving the provision of health services in village clinics. This study has substantiated the positive impact of the HAMs intervention on the number of visits.

Our findings demonstrated that the HAMs intervention program resulted in significant changes both in medical and pharmaceutical revenue. These changes included an increase of 54.03% in medical revenue and an increase of 32.84% in pharmaceutical revenue in the third phase for the HAMs intervention. This finding aligns with the existing literature, which suggested that SHSs improve access to healthcare. Several previous studies have demonstrated that telemedicine, as an expandable module of HAMs, can expand access to healthcare [27] and increase the revenue of health services [28–31]. We also observed that the HAMs intervention led to a significant increase by 2.368 CNY in medical expense per patient. These findings could be attributed to various factors facilitated by the HAMs intervention, such as improvement of the patient’s medical experience and the quality of healthcare services.

The study found that there were no significant changes in the level or slope of health services indicators during the second phase of intervention compared to the first phase. These findings are different from several other studies where the effects of SHS intervention became evident within six months post-introduction or even earlier [32, 33]. There are several potential explanations for this phenomenon. Firstly, the implementation of HAMs intervention across village clinics throughout the entire Hainan Province requires a longer time compared to its implementation in a single center. Secondly, for large-scale multi-center interventions, additional complexities arise that include gaps in policy-making, low technical literacy among users, and inadequate training for administrators and providers. Thirdly, village clinics in Hainan Province face an imbalance and scarcity of medical resources, characterized by a shortage of high-quality talents, an aging workforce of rural health workers, and inadequate service capability [34]. These factors may prolong the time required for rural health workers to become proficient in utilizing the functions of HAMs. Fourthly, the COVID-19 epidemic has exerted a discernible impact on the implementation of HAMs. Measures like blockade have restricted residents’ mobility, thereby impeding the promotion and utilization of HAMs. Moreover, rural healthcare workers have actively participated in epidemic prevention and control efforts, which have impeded the anticipated advancement of HAMs.

## Limitations

There were several limitations to this study. Firstly, the retrospective nature of the study design, combined with a before-after study design lacking a control group, precludes a direct causal interpretation of the results. There were other factors that might influence these changes. For example, other policy interventions that encouraged the use of the PHIS to record patient visit information and the phenomenon of medical inflation may also lead to an increase in medical expense. Secondly, due to the limited medical information technology in village clinics, certain patient data, such as individual patient data, patient demographics, and other relevant factors, remained inaccessible. It is not certain whether the HAMs intervention affected the quality of care for patients with chronic diseases who are expected to benefit from HAMs intervention. Thirdly, since the Hainan government launched the PHIS in primary medical institutions throughout the province beginning in 2020, we were unable to obtain earlier aggregated data related to outcome indicators before the HAMs intervention. This factor and other unmeasured time-varying confounders may have influenced our study. Finally, this study has limited generalizability because it was occurred within a health system of a small province in China. The findings of this study can only be reasonably generalized to other areas with similar contexts. It’s essential to note that our study included only the village clinics with records of patient visits in the PHIS, thus the findings of our study cannot be interpreted as the impact of the HAMs intervention on village clinics that rely on traditional paper prescriptions for recording patient visit information.

## Conclusions

In this study, we designed an ITS analysis using ARIMA models to analyze the effectiveness of the HAMs implementation in a large amount of village clinics. We analyzed the impact of the HAMs intervention on access to healthcare. Our research revealed that the HAMs intervention has led to significant improvements in various aspects of access to healthcare. The integration of multiple functions in the HAMs expanded the scope of healthcare services in rural areas, increasing both the number of patient visits and medical revenue. The HAMs have the potential to improve access to healthcare, but they require a certain period of adaptation for the target populations. The conclusions of this study have certain generalizability for medical institutions that share a similar context.

## Supplementary Information

Supplementary Material 1.

## Data Availability

No datasets were generated or analysed during the current study.

## Abbreviations

SHSs: Smart healthcare systems
AI: Artificial Intelligence
IoT: Internet of Things
HAMs: Health All-in-One Machines
ITS: Interrupted time series
ARIMA: Autoregressive integrated moving average
PHIS: Primary health information system
AIC: Akaike Information Criterion
MLE: Maximum Likelihood Estimation
ACF: Autocorrelation function
PACF: Partial autocorrelation function
AR: Autoregressive
CNY: Chinese Yuan
RV: Residual Variance
RMSE: Root Mean Squared Error
MAPE: Mean Absolute Percentage Error
