# Supplementary material for: Impact of Health All-in-One Machines on access to healthcare of rural areas in China: an interrupted time series analysis
Source: BMC Health Serv Res. 2025 Apr 12;25:537. doi: 10.1186/s12913-025-12710-z (PMC11992834; doi:10.1186/s12913-025-12710-z)
Supplement: Supplementary file 1 — Supplementary Material 1. [file 12913_2025_12710_MOESM1_ESM.docx]

**Supplementary Materials**

**The time series analyses and residual checks for the three outcome indicators**

**Fig. S1** Time series analysis of medical revenue: the time series of medical revenue (A), the residuals of segmented regression with no ARIMA errors modeling (B), autocorrelation function for the residuals (C), and partial autocorrelation function for the residuals (D)

**Fig. S2** Time series analysis of pharmaceutical revenue: the time series of pharmaceutical revenue (A), the residuals of segmented regression with no ARIMA errors modeling (B), autocorrelation function for the residuals(C), and partial autocorrelation function for the residuals (D)

**Fig. S3** Time series analysis of medical expense per patient: the time series of medical expense per patient (A), the residuals of segmented regression with no ARIMA errors modeling (B), autocorrelation function for the residuals(C), and partial autocorrelation function for the residuals (D)

**Fig. S4** Residual check of medical revenue for final ARIMA (1,0,0) model

**Fig. S5** Residual check of pharmaceutical revenue for final ARIMA (1,0,0) model

**Fig. S6** Residual check of medical expense per patient for final ARIMA (1,0,0) model

**Evaluation of the fit of ARIMA(1,0,0) error models for the first-phase and second-phase data**

Table S1 Fit measures of ARIMA(1,0,0) error models for the first-phase data

| Fit Measure | The number of patient visits | Medical revenue | Pharmaceutical revenue | Medical expense  per patient |
| --- | --- | --- | --- | --- |
| RV | 0.019 | 0.020 | 0.014 | 0.143 |
| RMSE | 0.130 | 0.132 | 0.109 | 0.348 |
| MAPE | 1.020 | 0.760 | 0.582 | 0.962 |

Note : RV: Residual Variance; RMSE: Root Mean Squared Error; MAPE: Mean Absolute Percentage Error.

Table S2 Fit measures of ARIMA(1,0,0) error models for the second-phase data

| Fit Measure | The number of patient visits | Medical revenue | Pharmaceutical revenue | Medical expense  per patient |
| --- | --- | --- | --- | --- |
| RV | 0.004 | 0.005 | 0.005 | 0.077 |
| RMSE | 0.063 | 0.064 | 0.067 | 0.260 |
| MAPE | 0.498 | 0.390 | 0.434 | 0.687 |

Note : RV: Residual Variance; RMSE: Root Mean Squared Error; MAPE: Mean Absolute Percentage Error.
